# Treatment patterns, adverse events, and economic burden in a privately insured population of patients with chronic lymphocytic leukemia in the United States

**DOI:** 10.1002/cam4.2268

**Published:** 2019-05-29

**Authors:** Shaum M. Kabadi, Ravi K. Goyal, Saurabh P. Nagar, James A. Kaye, Keith L. Davis

**Affiliations:** ^1^ AstraZeneca Gaithersburg Maryland; ^2^ RTI Health Solutions Research Triangle Park North Carolina; ^3^ RTI Health Solutions Waltham Massachusetts

**Keywords:** adverse events, chronic lymphocytic leukemia (CLL), economic burden, treatment patterns

## Abstract

**Introduction:**

Contemporary data describing treatment patterns, adverse events (AEs), and outcomes in patients with chronic lymphocytic leukemia (CLL) in clinical practice are lacking. We conducted a retrospective cohort study and assessed treatment patterns, AEs, health‐care resource use (HCRU), and costs in patients with diagnosis of CLL.

**Methods:**

Using a nationally representative population of privately insured patients in the US, adult patients with CLL diagnosis (July 2012‐June 2015) were selected if they had continuous health plan enrollment for ≥12 months before the first CLL diagnosis without any evidence of any CLL‐directed treatment. Treatment patterns up to four lines of therapy (LOT) and occurrence of AEs during CLL therapies were assessed. Mean per‐patient monthly HCRU and costs were assessed overall and by number of unique AEs.

**Results:**

Of all patients meeting the selection criteria (n = 7,639; median age, 66 years), 18% (n = 1,379) received a systemic therapy during study follow‐up. Of these, bendamustine/rituximab (BR) was the most common first observed regimen (28.1%), while ibrutinib was the most common therapy in the second (20.8%) and third (25.5%) observed regimens. The mean monthly all‐cause and CLL‐related costs, among patients treated with a systemic therapy, were $7,943 (SD = $15,757) and $5,185 (SD = $9,935), respectively. Mean monthly all‐cause costs increased by the number of AEs (from $905 [SD = $1,865] among those with no AEs to $6,032 [SD = $13,290] among those with ≥6 AEs).

**Conclusions:**

Chemoimmunotherapy, particularly BR, was the most common first observed therapy for CLL, whereas ibrutinib was most preferred in the second and third observed lines of therapy during the study period. Findings demonstrate that the economic burden of AEs in CLL is substantial.

## 1 INTRODUCTION

Chronic lymphocytic leukemia (CLL) is the most common type of leukemia, representing approximately one‐third (32%) of all newly diagnosed leukemia cases in the US.^1^ Small lymphocytic lymphoma (SLL) is like CLL in terms of the biologic characteristics of neoplastic lymphocytes and clinical behavior. Reported 5‐year median survival among patients with CLL (range, 8‐12 years)^2^, ^3^ has improved substantially over the past few decades, from 67.5% in 1975 to 87% in 2010.^4^

Treatment recommendations for patients with CLL, in the 2017 NCCN Clinical Practice Guidelines in Oncology (NCCN Guidelines^®^), are based on the disease state, the presence or absence of genetic abnormalities (especially p53 aberration—either by point mutation or deletion of chromosome 17p [del(17p)]—and mutation of the immunoglobulin heavy chain variable gene [*IGHV*]), and the patient's age and general health.^5^, ^6^ Currently available treatment options for CLL include chemoimmunotherapy (combinations such as fludarabine, cyclophosphamide, and rituximab [FCR] and bendamustine plus rituximab [BR]), or targeted therapies, specifically ibrutinib (a Bruton's tyrosine kinase inhibitor), idelalisib (an inhibitor of phosphatidylinositol 3‐kinase delta), and venetoclax (which inhibits an apoptosis‐suppressing oncogene, B‐cell leukemia/lymphoma 2), alone or in combination with rituximab. Newer anti‐CD20 antibodies similar to rituximab, such as ofatumumab or obinutuzumab, are also presently available.^6^

In the 2015 NCCN Guidelines, the recommended treatments for elderly patients (>70 years) with newly diagnosed CLL and significant comorbidities included obinutuzumab plus chlorambucil or rituximab (alone or in combination with chlorambucil) mainly due to limited ability to tolerate the purine analogues (eg, fludarabine) used in chemoimmunotherapy regimens.^7^ Those without significant comorbidities were recommended chemoimmunotherapy (FCR/FR/PCR/BR) or obinutuzumab plus chlorambucil.^7^ First‐line chemoimmunotherapy is also recommended currently for younger, fit patients with mutated *IGHV* and without del(17p) because of the excellent long‐term prognosis in this group.^6^ First‐line therapy with ibrutinib is also now approved for treatment of CLL in patients of all ages with or without del(17p).^8^

In 2015 guidelines, the recommendations for patients with relapsed or refractory disease and without significant comorbidity have been ibrutinib, idelalisib with or without rituximab, chemoimmunotherapy, ofatumumab, obinutuzumab, lenalidomide or alemtuzumab with or without rituximab, or high dose methylprednisolone and rituximab.^7^ Venetoclax was initially approved in April 2016 for treatment of patients with CLL with del(17p) who have received at least one prior therapy.^9^

Patients receiving CLL treatment may experience a range of mild to severe hematologic and nonhematologic adverse events (AEs). AEs can be a nuisance to patients and moderate to severe AEs may lead to treatment changes which may lower the quality of life and increase economic burden related to their management. At least four observational studies have been examined, namely treatment characteristics, AEs, health‐care resource use (HCRU) and costs in patients with CLL,^10^, ^11^, ^12^, ^13^ but they have mostly been limited to subgroups of patients such as those receiving a specific treatment^13^ or those treated at selected institutions.^11^ Therefore, in this study we aimed to conduct a detailed assessment of treatment patterns, AEs, HCRU, and direct health‐care costs in a nationally representative group of privately insured patients with a diagnosis of CLL in the US.

## 2 METHODS

### 2.1 Design and data source

In this retrospective cohort study, the IBM MarketScan Research Databases containing administrative claims data for a large, nationally representative sample of individuals in employer‐sponsored private health insurance plans across the US were used. These databases provide longitudinal data on medical and pharmacy service utilization and associated payments, collected from nearly 350 employers and payers in the US. They contain health‐care information for employed individuals and their dependents covered under fee‐for‐service and various capitated health plans. Patient data for each health‐care encounter and associated diagnoses and treatments, as recorded in claims forms using applicable coding, are recorded. Payments and charges including amounts paid by the health plan and the amount of patient responsibility are also captured. IBM MarketScan databases have been widely used for conducting retrospective observational studies of health outcomes in the US, such as this one, with more than 1,000 overall publications in peer‐reviewed journals.^14^, ^15^, ^16^, ^17^, ^18^

This database does not represent individuals who are enrolled only in a public health insurance program (eg, Medicare, Medicaid) with no supplemental private insurance or those who are unemployed and/or uninsured. Also, there are no restrictions based on age or economic status; however, because the database captures information on individuals employed with private insurance (or Medicare with a supplemental insurance), the population tends to be economically superior to those not represented in the data.

As the study data were retrospective, de‐identified, and anonymous, RTI International's institutional review board committee determined that this study does not constitute research with human subjects and was therefore exempt from institutional review board consideration.

### 2.2 Patient selection

**Figure 1:**
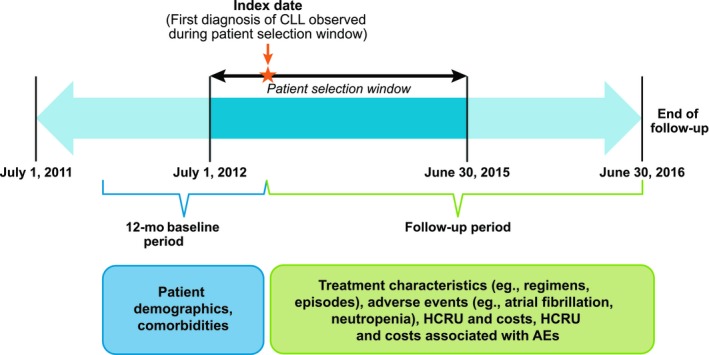
Graphical summary of the study design. AE, adverse event; CLL, chronic lymphocytic leukemia; HCRU, health‐care resource use

Patients with CLL were identified using diagnostic codes 204.1x (ICD‐9‐CM) and C91.1x (ICD‐10‐CM) during the period 1 July 2012‐30 June 2015. Patients with SLL (C83.0x [ICD‐10‐CM]) were considered part of the CLL population and were included in the analysis. Patients were required to have ≥2 medical claims on separate dates with diagnosis code(s) for CLL, where the date of the first medical claim defined the study index date. Eligible patients were also aged ≥18 years at the index date; had at least 12 months of continuous enrollment (with gaps ≤30 days permitted) in medical and drug plans, with no capitation, before the study index date; and had no evidence of CLL or CLL‐directed treatment (systemic therapy and/or stem cell transplant (SCT)) during the 12‐month pre‐index date (baseline) period. All patients were followed through the earlier of disenrollment from the medical and/or drug plan or end of the study period (30 June 2016). A summary of the study design is presented in Figure 1.

### 2.3 Study measures

#### 2.3.1 Patient characteristics

Patient demographic and other baseline characteristics, including Charlson Comorbidity Index (CCI) score, were assessed at index or during the 12‐month baseline period.^19^ All comorbidities were assessed during the 12‐month period on or before the index CLL diagnosis date). In addition, baseline atrial fibrillation (A‐fib) risk status (high vs low) was defined based on the assessment of seven risk factors: heart failure, hypertension, diabetes, age 65‐74 years (at index), age ≥75 years (at index), coronary artery disease, and chronic kidney disease.^20^ Patients with evidence of at least one of the following were classified as “high‐risk” A‐fib patients: (a) any two of the first five risk factors listed above, (b) any three of all seven risk factors listed above, or (c) history of A‐fib during the baseline period.

#### 2.3.2 Therapy regimens

CLL‐directed treatments recommended in the 2017 NCCN Guidelines^®^ were identified using applicable procedure and medication codes and defined based on the criteria presented in the Supplementary Table A1.^5^ This study focused on patients treated with systemic therapy regimens. Some patients had evidence of chemotherapy receipt as per the recorded “general administration” codes but did not have any other treatment codes identifying specific agent(s) administered; these patients were not included in the assessment of observed therapy regimens due to lack of specificity. Detailed characteristics of each treatment regimen, including composition, time to initiation, and duration were assessed for up to four lines of therapy (LOTs). Lines of therapy were reported based on observed regimens because it was not known if the patient received treatment in a frontline versus relapsed/refractory setting, since the baseline period was limited to 12 months in this study.

#### 2.3.3 Adverse events

AEs were identified as medical claims containing ICD‐9‐CM/ICD‐10‐CM diagnosis codes for specific AEs (at any position). AEs commonly reported in previous studies as associated with treatments for CLL were selected. The first occurrence of an AE during a CLL treatment defined the incidence of a potentially treatment‐related AE. For selected AEs related to chronic disease (A‐fib and hypertension), evidence of a prior history precluded patients from being considered at risk of the event during a CLL treatment.

#### 2.3.4 Health‐care resource use and costs

CLL‐related and all‐cause HCRU and costs were assessed during the post‐index date period. Cost data were adjusted to 2016 US dollars using the medical care component of the US Consumer Price Index and included health plan paid amounts and the coordination of benefit amounts. HCRU and cost measures were reported by care setting (ie, inpatient, emergency department, outpatient hospital, physician office, hospice, pharmacy, and ancillary). In addition, costs associated with specific CLL treatments were assessed using all medical and pharmacy costs incurred during the first episode of a given treatment (regardless of the LOT on which the treatment was initiated).

### 2.4 Data analysis

Descriptive analyses are reported for all study measures. Mean per‐patient per month HCRU and costs were assessed to allow for variable length of follow‐up. The total number of unique AEs recorded per‐patient during the follow‐up period was tabulated (categorized as 0, 1‐2, 3‐5, or ≥6 AEs). The incremental HCRU and costs associated with AEs were analyzed by stratifying these measures by the number of AEs experienced using the categorization specified above.

A multivariable logistic regression model was performed to assess factors associated with inpatient admission in the first LOT (LOT‐1), and generalized linear models were fit to assess factors predictive of health‐care costs in LOT‐1. A binary variable representing the number of unique AEs observed during LOT‐1 (0‐2, 3‐5, and ≥6 AEs) was included in the model to assess incremental inpatient admission and cost burdens associated with number of AEs. All analyses were performed using SAS statistical software, version 9.4 (SAS Institute Inc; Cary, NC; 2011).

## 3 RESULTS

### 3.1 Baseline patient characteristics

A total of 7,639 patients with CLL met the selection criteria (median age, 66 years [range, 18‐100]; 58% male). The sample selection flowchart is presented in Supplementary Figure A1. The mean CCI score in the 12‐month baseline period was 2.1 (range, 0‐15), Among clinical conditions related to CLL at baseline, the most common were infection (49%), hypertension (40%), dyspnea (35%), arthralgia (22%), anemia (21%), fatigue/asthenia (18%), and hemorrhage (16%). The mean monthly all‐cause cost during the baseline period was $962 (standard deviation [SD] = $2,980). Baseline patient characteristics are presented in Table 1.^19^

**Table 1:** Baseline characteristics of patients with CLL

All Patients, n (%)	7,639 (100.0%)
Age at index, years	
Mean (SD)	67.6 (12.7)
Median (Q1, Q3)	66 (59, 78)
Health plan type, n (%)	
HMO	776 (10.2%)
PPO	3,972 (52.0%)
POS	434 (5.7%)
Other	2,310 (30.2%)
Unknown	147 (1.9%)
Year of study index date (first diagnosis), n (%)	
2012	1,825^e^ (23.9%)
2013	2,635 (34.5%)
2014	2,281 (29.9%)
2015	898^e^ (11.8%)
Length of follow‐up (months)^a^	
Mean (SD)	22.0 (12.8)
Median	20.6
Min, Max	0.1, 47.9
Atrial fibrillation risk status^b^, n (%)	
High risk	3,565 (46.7%)
Low risk	4,074 (53.3%)
CCI score	
Mean (SD)	2.1 (2.3)
Median (Q1, Q3)	1 (0, 3)
Min, Max	0, 15
Daily pill burden^c^	
Mean (SD)	2.5 (2.8)
Median (Q1, Q3)	2 (0, 4)
Min, Max	0, 27
Average monthly costs^d^	
Mean (SD)	$962 ($2,980)
Median (Q1, Q3)	$325 ($135, $752)
Min, Max	$0, $103,582

Note

All costs are in 2016 US dollars.

Abbreviations: CCI, Charlson Comorbidity Index; CLL, chronic lymphocytic lymphoma; HMO, health maintenance organization; POS, point of service; PPO, preferred provider organization; Q1, first quartile; Q3, third quartile; SD, standard deviation.

a Follow‐up time calculated as the number of days between the study index date and the end of the follow‐up divided by 30.5.

b Atrial fibrillation risk status was defined based on the method used by Chyou et al.

c Mean number of oral medications available in hand, on a daily basis, during the 30‐day period before the study index date.

d Mean monthly all‐cause costs over the 12‐month baseline period (includes costs for inpatient stays, emergency department visits, office visits, other outpatient and ancillary care, and pharmacy visits) as incurred by health plans.

e Indicates data for partial year: for 2012, data include diagnoses from July through December, and for 2015, data include diagnoses from January through June.

### 3.2 Treatments and adverse events

Patients had a median 20.6 months (IQR = 12.4‐31.5) follow‐up after their first CLL diagnosis. Twenty‐nine percent (n = 2,211) of patients received at least one CLL‐directed treatment; of these, nearly 62% (n = 1,379) initiated treatment with a systemic therapy (ie, LOT‐1) that was identifiable using the agent‐specific treatment codes available in the claims data. Overall, among patients treated with a prior LOT, 26% (n = 355) received LOT‐2, 30% (n = 106) received LOT‐3, and 33% (n = 35) received LOT‐4, during follow‐up. The most common systemic therapy regimens, regardless of observed therapy line, were BR (32%), rituximab monotherapy (24% [including maintenance]), ibrutinib monotherapy (15%), and FCR (14%). The most common LOT‐1 regimen was BR (28.1%), while ibrutinib was the most common regimen in LOT‐2 (20.8%) and in LOT‐3 (25.5%). Use of idelalisib was limited to 1.6% of all patients receiving systemic therapy; however, an increasing trend was observed as patients moved from first to fourth LOT (<1% in LOT‐1, 3.1% in LOT‐2, 4.7% in LOT‐3, and 8.6% in LOT‐4). In patients aged ≥65 years, 5% received FCR as LOT‐1 (vs 20% in patients aged 18‐64 years). In contrast, rituximab monotherapy was used in 26% of patients aged ≥65 years (vs 15% in those aged 18‐64 years).

The median times to initiation of treatment from the study index date for the most common regimens were 16.4 months (IQR = 8.5‐23.9) for ibrutinib monotherapy, 7.7 months (IQR = 1.6‐16.9) for rituximab monotherapy, 3.3 months (IQR = 0.9‐13.3) for BR, and 2.4 months (IQR = 0.9‐10.4) for FCR. The median durations of exposure were 7.5 months (IQR = 4.1‐14.2) (ibrutinib monotherapy), 4.7 months (IQR = 2.9‐5.8) (BR), 1.7 months (IQR = 1.6‐3.3) (rituximab monotherapy), and 4.7 months (IQR = 2.9‐5.8) (FCR).

The most common AEs (≥10% in at least one of the treatment regimens) observed during CLL treatments are shown in Table 2. The incidences of neutropenia, dehydration, fatigue/asthenia, and nausea were higher among those receiving chemoimmunotherapy than those who received ibrutinib monotherapy. Conversely, A‐fib, hypertension, hemorrhage/bleeding, and pneumonia were more common among patients receiving ibrutinib monotherapy than those who received chemoimmunotherapy.

**Table 2:** Adverse events identified during treatments for chronic lymphocytic leukemia

	BR	FCR	Rituximab Monotherapy	Ibrutinib Monotherapy
	(n = 446)	(n = 194)	(n = 327)	(n = 201)
Hematologic adverse events				
Anemia	35%	32%	37%	35%
Thrombocytopenia	16%	17%	19%	20%
Neutropenia	58%	72%	6%	12%
Nonhematologic adverse events				
Atrial fibrillation^a^	2%	3%	3%	11%
Dehydration	15%	15%	7%	8%
Dyspnea	28%	24%	19%	25%
Fatigue/asthenia	18%	18%	10%	12%
Fever/pyrexia	17%	13%	6%	12%
Hemorrhage/bleeding	7%	7%	9%	13%
Hypertension^a^	2%	2%	3%	13%
Infection	36%	21%	28%	38%
Nausea/vomiting	32%	34%	13%	6%
Pneumonia	7%	6%	8%	12%

Note

Data reported for the most common adverse events (≥10% in at least one of the columns).

Abbreviations: BR, bendamustine/rituximab; FCR, fludarabine, cyclophosphamide, and rituximab.

a Baseline history of the adverse event precluded patients from being considered at risk for that adverse event during the follow‐up period.

### 3.3 Resource use and costs

**Figure 2:**
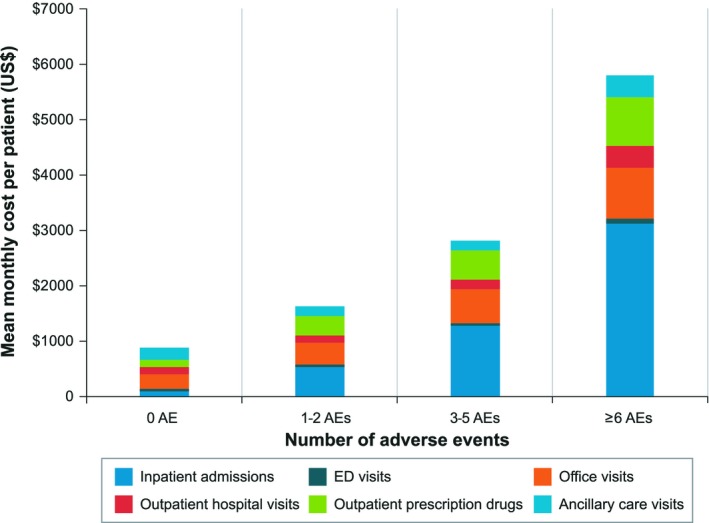
All‐cause monthly health‐care costs by practice setting and number of AEs

Mean monthly per‐patient costs during the post‐index date follow‐up, among all patients, were $3,784 (SD = $10,244) for all‐cause and $1,885 (SD = $6,438) for CLL‐related events. Among patients treated with a systemic therapy (n = 1,379), the mean monthly all‐cause and CLL‐related costs were $7,943 (SD = $15,757) and $5,185 (SD = $9,935), respectively. Figure 2 depicts mean monthly costs by care setting and number of AEs, among all patients. Mean (SD) monthly per‐patient costs during individual treatments were as follows: $14,640 ($12,925) (BR), $12,575 ($18,072) (rituximab monotherapy), $21,766 ($36,140) (ibrutinib monotherapy), and $12,742 ($15,994) (FCR).

When the HCRU was stratified by number of AEs, inpatient admissions per‐patient increased from 3.4% among those with no AE to 66.1% among those with ≥6 AEs. The mean length of stay per admission also increased with number of AEs: 2.4 days (0 AEs), 4.4 days (1‐2 AEs), 5.0 days (3‐5 AEs), and 5.7 days (≥6 AEs). Mean (SD) monthly all‐cause costs during the post‐index date follow‐up were $905 ($1,865) among those with no AEs, $1,655 ($5,364) among those with 1‐2 AEs, $2,883 ($8,483) among those with 3‐5 AEs, and $6,032 ($13,290) among those with ≥6 AEs.

### 3.4 Factors associated with inpatient admission and costs

Patients experiencing 3‐5 AEs during LOT‐1 had nearly seven times greater odds of an inpatient admission in LOT‐1 than those experiencing 0‐2 AEs (OR = 6.96; 95% CI, 4.63‐10.48). Among those with ≥6 AEs, the odds of an inpatient admission during LOT‐1 were 22 times higher than those with 0‐2 AEs (OR = 22.27; 95% CI, 14.19‐34.95).

Occurrences of anemia (cost ratio [CR] = 1.70; 95% CI, 1.48‐1.96), infection (CR = 1.17; 95% CI, 1.0‐1.36), neutropenia (CR = 1.18; 95% CI, 1.02‐1.37), and pneumonia (CR = 1.32; 95% CI, 1.02‐1.72) were associated with significantly higher monthly all‐cause costs (compared with absence of the respective AE) during LOT‐1. First LOT with FCR (vs BR) was associated with lower monthly all‐cause costs (CR = 0.74; 95% CI, 0.60‐0.93), while ibrutinib monotherapy was associated with 59% higher monthly all‐cause costs (CR, 1.59; 95% CI, 1.19‐2.13).

## 4 DISCUSSION

This population‐based study yielded recent real‐world evidence on treatment patterns, AEs, HCRU, and costs in patients enrolled in health plans in the US. Our findings set a comprehensive benchmark against which the future therapeutic landscape can be compared. Since our study population was derived from active employees or early retirees, they were relatively younger (half were <66 years of age) and less frail. The finding that nearly two‐thirds of patients did not receive a cancer‐directed therapy after CLL diagnosis suggests that observation (“watch‐and‐wait”) was the preferred initial approach during the study period. Our study identified BR, rituximab monotherapy, ibrutinib monotherapy, and FCR as the most common regimens during the study period, which is consistent with the 2017 NCCN Guidelines and other recently published reports.^5^, ^21^, ^22^ Recently, 2018 NCCN Guidelines suggest chemoimmunotherapy (FCR/ BR) or ibrutinib as preferred treatment choices among younger patients (<65 years) who are relatively less frail and able to tolerate purine analogues.^23^ The most common choice among LOT‐1 regimens was BR, while ibrutinib was the preferred LOT‐2 regimen, a finding that confirms results of another study^21^; however, the proportion of ibrutinib users observed in our study was half of what has been reported (21% vs 42%), potentially because in our analysis ibrutinib was approved only in the later part of the study period. Ibrutinib uptake is expected to increase over the next decade.^21^, ^24^

The frequencies of hematologic AEs in this study were generally similar to those in previously published reports of CLL treatments, but the frequencies of nonhematologic AEs were generally higher than those in other published studies involving chemoimmunotherapy regimens.^12^, ^25^, ^26^, ^27^ The finding that the mean monthly per‐patient costs increased gradually with the number of unique AEs highlights the substantial economic burden associated with toxicities. Because fewer than only one‐third of patients received a CLL‐directed treatment during the observation period, it is also likely that the increases in number of AEs and associated costs are correlated with the receipt as well as the nature of CLL‐directed therapies.

Findings from this study are subject to limitations pertaining to the data source and the study design and should be interpreted within this context. First, selection of the study cohort was based on diagnosis codes indicative of CLL as recorded in insurance claims. Any erroneous use of diagnostic code may lead to misclassification of patients with CLL. Second, patients' medical records were not available, and it was assumed that claims associated with AEs were accurately coded and that the costs associated with claims are legitimately related to CLL treatment or CLL complications as applicable. Furthermore, our data source does not contain information on all prognostic factors (eg, del[17p] status, TP53 mutation status, complex karyotype, IGHV mutation), which could have influenced treatment patterns and outcomes. Finally, a 12‐month “clean” period before the first recorded diagnosis of CLL was required to identify patients with “new” CLL diagnosis, but the study population still could have included some patients who were previously diagnosed and had not had medical encounters related to CLL for more than 12 months.

## 5 CONCLUSIONS

Consistent with the NCCN Guidelines during the study period, chemoimmunotherapy, particularly BR, was the most common first observed therapy, whereas ibrutinib was most preferred in the second and third observed therapy lines. The findings indicate that resource utilization and economic burden of CLL is substantial, with monthly per‐patient costs varying considerably by care setting, treatment regimen, and number of AEs. Patients frequently experienced hematologic and nonhematologic AEs, and their incidence varied by type of treatment. This study shows that the AE burden associated with current treatments for CLL is substantial, and the management of AEs occurring during treatments may have a significant impact on overall health‐care costs.

## DISCLOSURE STATEMENT

Shaum M. Kabadi is a full‐time employee of AstraZeneca, the funding organization. Ravi K. Goyal, Saurabh P. Nagar, Keith L. Davis, and James A. Kaye are full‐time employees of RTI Health Solutions, which received funding from AstraZeneca to conduct this research.

## Data Availability

The data that support the findings of this study are available from a third‐party, IBM. Restrictions apply to the availability of these data, which were used under license for this study.
