# Supplementary material for: Treatment patterns, adverse events, and economic burden in a privately insured population of patients with chronic lymphocytic leukemia in the United States
Source: Cancer Med. 2019 May 29;8(8):3803–10. doi: 10.1002/cam4.2268 (PMC6639180; doi:10.1002/cam4.2268)
Supplement: Supplementary file 1 [file CAM4-8-3803-s001.docx]

Figure A1. Sample Attrition Flowchart for Patients With Chronic Lymphocytic Leukemia (Online Only)

ICD-9-CM = International Classification of Diseases, Ninth Revision, Clinical Modification; CLL = chronic lymphocytic leukemia; MCL = mantle cell lymphoma.

^a^CLL-directed treatment exclusion includes chemotherapy, biologic therapy, targeted therapy, immunotherapy, and stem cell transplant.

^b^Study index date represents the date of first CLL diagnosis observed between July 1, 2012, and June 30, 2015, inclusive, among patients meeting eligibility criteria.

Table A1. Selected Study Outcomes Definitions (Online Only)

| Outcome | Definition |
| --- | --- |
| Atrial fibrillation (A-fib) risk status | Based on assessment of seven risk factors: heart failure, hypertension, diabetes, age 65-74 years (at index), age ≥75 years (at index), coronary artery disease, and chronic kidney disease. Patients with evidence of at least one of the following were classified as “high-risk” A-fib patients: (1) any two of the first five risk factors listed above, (2) any three of all seven risk factors listed above, or (3) history of A-fib during the baseline period. |
| CLL-directed treatment regimens | The first-line systemic therapy regimen was defined as the combination of all agents observed on or within 35 days after the first claim for a systemic therapy drug following the study index date (claims with generic chemotherapy encounter/administration codes were excluded). For parenteral drugs, the first-line therapy (LOT-1) ended 30 days after the last administration. For oral medications (e.g., ibrutinib), the prescription days’ supply was used to determine the duration of treatment. The date of the end of days’ supply based on the last observed refill of the oral regimen, with subsequent treatment gap ≥90 days, defined the end of LOT-1. The end of the therapy line for oral regimens was calculated by adding the total number of days’ supply to the prescription fill date plus the allowed gap of 90 days. Patients who switched regimens, with or without a 90-day gap in treatment, were considered as having initiated a new LOT. Maintenance therapy with rituximab was defined as rituximab monotherapy initiated within 7 months after completion of a rituximab-containing combination therapy (e.g., rituximab/cyclophosphamide/doxorubicin/vincristine [RCHOP], with or without prednisone). A >7- month gap after the last administration of rituximab defined the end of rituximab maintenance therapy. |
